# Individual responsiveness of macrophage migration inhibitory factor predicts long-term cognitive impairment after bacterial meningitis

**DOI:** 10.1186/s40478-020-01100-7

**Published:** 2021-01-06

**Authors:** Anne T. Kloek, Mercedes Valls Seron, Ben Schmand, Michael W. T. Tanck, Arie van der Ende, Matthijs C. Brouwer, Diederik van de Beek

**Affiliations:** 1 Department of Neurology, Amsterdam UMC, University of Amsterdam, Amsterdam Neuroscience, Meibergdreef 9, 1105 AZ Amsterdam, The Netherlands; 2 Department of Medical Psychology, Amsterdam UMC, University of Amsterdam, Amsterdam Neuroscience, Meibergdreef 9, 1105 AZ Amsterdam, The Netherlands; 3 Department of Psychology, Brain and Cognition, University of Amsterdam, Nieuwe Achtergracht 129 B, 1001 NK Amsterdam, The Netherlands; 4 Department of Epidemiology and Data Science, Amsterdam UMC, University of Amsterdam, Amsterdam Public Health, Meibergdreef 9, 1105 AZ Amsterdam, The Netherlands; 5 Department of Medical Microbiology and Infection Prevention, Amsterdam UMC, University of Amsterdam, Amsterdam Infection and Immunity, Meibergdreef 9, 1105 AZ Amsterdam, The Netherlands; 6 Department of Medical Microbiology and Infection Prevention, The Netherlands Reference Laboratory for Bacterial Meningitis, Amsterdam Infection and Immunity, Meibergdreef 9, 1105 AZ Amsterdam, The Netherlands; 7 Department of Neurology, Amsterdam UMC, University of Amsterdam, Amsterdam Neuroscience, PO Box 22660, 1100DD Amsterdam, The Netherlands

**Keywords:** Macrophage migration inhibitory factor, Bacterial meningitis, Pneumococcal meningitis, Cognitive impairment

## Abstract

**Background:**

Patients with pneumococcal meningitis are at risk for death and neurological sequelae including cognitive impairment. Functional genetic polymorphisms of macrophage migration inhibitory factor (MIF) alleles have shown to predict mortality of pneumococcal meningitis.

**Methods:**

We investigated whether MIF concentrations during the acute phase of disease were predictive for death in a nationwide prospective cohort study. Subsequently, we studied whether individual ex vivo MIF response years after meningitis was associated with the development of cognitive impairment.

**Results:**

We found that in the acute illness of pneumococcal meningitis, higher plasma MIF concentrations were predictive for mortality (*p* = 0.009). Cognitive impairment, examined 1–5 years after meningitis, was present in 11 of 79 patients after pneumococcal meningitis (14%), as compared to 1 of 63 (2%) in controls, and was consistently associated with individual variability in MIF production by peripheral blood mononuclear cells after ex vivo stimulation with various infectious stimuli.

**Conclusions:**

Our study confirms the role of MIF in poor disease outcome of pneumococcal meningitis. Inter-individual differences in MIF production were associated with long-term cognitive impairment years after pneumococcal meningitis. The present study provides evidence that MIF mediates long-term cognitive impairment in bacterial meningitis survivors and suggests a potential role for MIF as a target of immune-modulating adjunctive therapy.

## Background

Acute bacterial meningitis is a life-threatening disease that ranks among the top 10 infectious causes of death [1]. *Streptococcus pneumoniae* is the most common cause of bacterial meningitis, accounting for 75% of cases in developed countries [2, 3]. Pneumococcal meningitis is associated with mortality ranging from 6 to 24% and a substantial morbidity ranging from 23–29% [3–8]. Common neurological sequelae after pneumococcal meningitis are focal cerebral deficits (11–36%), hearing loss (22–69%) and, seizures (4–31%) [4, 9–12].

Cognitive impairment occurs in 14–32% after pneumococcal meningitis and even in those with apparent good clinical outcome [13–15]. A long-term follow up of adults with bacterial meningitis included in a randomized controlled study on the adjunctive dexamethasone therapy, showed no difference in neuropsychological outcome between dexamethasone- and placebo treated patients [16]. However, in a cross-sectional study, we recently described that pneumococcal meningitis patients treated with dexamethasone had less frequent cognitive impairment compared to patients not treated with dexamethasone [15].

Macrophage migration inhibitory factor (MIF) plays an important role in our innate immune system as a pro-inflammatory cytokine and a neuro-endocrine mediator [17, 18]. MIF is expressed by cells of the immune system but also by cells of the central nervous system and various other organs [19]. It stimulates cytokine production of macrophages and enhances Toll-like receptor 4 expression on macrophage surface increasing phagocytosis and inhibiting apoptosis [17]. It also acts as an endogenous counter-regulator of glucocorticoid immunosuppressive action [18]. MIF has been implicated as playing a causative role in many disease states, including sepsis, pneumonia, diabetes, rheumatoid arthritis, inflammatory bowel disease, cancer, and inflammatory skin disease [20]. MIF also has been associated with the development of cognitive impairment, mainly in Alzheimer’s disease [20]. We previously have identified MIF as genetic marker of patient’s outcome in community-acquired bacterial meningitis [21]. In a prospective, nationwide cohort of patients with pneumococcal meningitis, we showed high-expression MIF alleles were associated with disease severity and death [21]. In patients with pneumococcal meningitis MIF cerebrospinal fluid (CSF) values were increased and high CSF MIF levels are associated with systemic complications and death [21, 22].

Here, we further define the role of MIF on outcome in pneumococcal meningitis. We investigated associations between serial MIF blood levels and outcome in the acute illness. Furthermore, we evaluated the long-term cognitive outcome of these patients and determined associations between the inter-individual variability of MIF concentrations after ex vivo stimulatory experiments and cognitive impairment.

## Methods

### Serial blood sampling in bacterial meningitis patients

Patients with bacterial meningitis admitted between April 2014 and January 2017 in one of our 12 participating centers in the Netherlands were included. Inclusion criteria were a clinical suspicion on bacterial meningitis and one of the following CSF characteristics: pleiocytosis > 1000 cells per 3 mm^3^, glucose < 1.9 mmol/L, protein > 2.20 g/L or a positive Gram stain. Bacterial meningitis needed to be confirmed with either a positive CSF culture of positive blood culture. Blood samples were withdrawn on day 0, 1, 2 and 7 of admission and 3 months after discharge. Blood samples were immediately processed in the participating hospitals and stored at − 70 or − 80 degrees ºC. Patients in this study were included in the MeninGene study as well, a nation-wide Dutch prospective cohort study analyzing genetic risk factors in bacterial meningitis, described elsewhere [3]. Clinical data and outcome were prospectively collected by the attending physicians, mostly neurologists, in an online database. Outcome was scored at discharge by the Glasgow Outcome Scale (GOS) score, a score ranging from 1 to 5, a score of 1 indicating death, 2 is persistent vegetative state, 3 is severe disability, 4 is moderate disability (capable of living independently but unable to return to work or school) and, 5 is mild or no disability (able to return to work or school) [23]. Patients were included during the acute phase of the illness and provided written informed consent for participation for both the MeninGene and the serial sampling study.

### Patient cohort and controls of recall study

Participants in the recall study had been included in the MeninGene study between October 2011 and March 2015. Patients in this study were older than 16 years of age and had a community-acquired acute bacterial meningitis confirmed by CSF cultures, or a positive CSF PCR in combination with typical CSF abnormalities. On the informed consent form of the MeninGene study, the patient was asked whether they allowed the researchers to approach them for follow-up studies on long-term neurological sequelae. Patients eligible for the current follow-up study provided this consent and had been admitted with pneumococcal meningitis 1–5 years prior the follow-up study. The control group consisted of the partners or other proxies of the patients. Before participation patients and controls were questioned about their medical history, medication use, and ongoing illness. If patients had ongoing infections or felt ill they could not participate in the study. Patients who gave permission to participate in this follow-up study were recalled to the Academic Medical Center for a blood withdrawal and neuropsychological examination.

### Whole blood and PBMC stimulation experiments

In the Recall study blood from patients and controls was collected in heparin tubes. To isolate peripheral blood mononuclear cells (PBMCs) the whole blood was 1:1 diluted with Dulbecco's phosphate-buffered saline (D-PBS) and thereafter centrifuged with Ficoll®. Isolated PBMCs were washed three times with D-PBS before diluted in Roswell Park Memorial Institute (RPMI) medium. Whole blood and isolated PBMCs were stimulated at 37 ºC with RPMI, lipopolysaccharide (LPS) 10 ng/ml, lipoteichoic acid (LTA) 1000 ng/ml and ultraviolet (UV) killed *S. pneumoniae* strains D39 (serotype 2) multiplicity of infection (MOI) 10 and 6303 (serotype 3) MOI 5. After 24 h stimulated samples were centrifuged for 10 min at 400 × G and supernatant was collected and stored at − 80 ºC until further use.

### Cytokine measurements

Human MIF, IL-6 and IL-10 levels in the blood samples of the serial sampling and the stimulation experiments were measured with the Luminex® technology by using an assay of Bio-Techne. Measurements were done according manufacturing protocol. The lower and upper limit of detection for MIF were respectively 219 pg/ml and 55,600 pg/ml.

### Neuropsychological examination

Cognitive functioning was tested with the Cognitive Basic Assessment Test set (COGBAT) of the Vienna Test System (VTS), Schuhfried, Mödling, Austria. Details of this test set are previous described [15]. All VTS COGBAT normative test scores were expressed as z-scores corrected for age and education with the control group as a reference. The value of the z-score represents the distance between the patient score and the mean control group score, in units of the standard deviation. The z-score is negative when the patient score is below the mean control group score and positive when above. To compare differences in MIF concentration between groups with worse versus good scores on cognitive performance, the groups were divided in < − 1 SD (z score < − 1, worse scores) versus ≥ − 1 SD (z score ≥ − 1, good scores).

### Statistical analyses

Data was analyzed by using IBM SPSS statistics (version 24). Differences in MIF concentration between groups were calculated with the t test or Mann Whitney U test depending respectively on a normal or skewed distribution. If not normal distributed, MIF concentrations in the stimulation experiments were converted to a normal distribution with a log transformation. The Friedman test was used to compare paired samples in all groups (> 2) and the Wilcoxon signed rank test was used to compare two related samples. All tests were two-tailed and *p* values of < 0.05 were considered as statistical significant. Statistical analyses to examine difference in cognitive functioning between patients and controls are described in a previous study [15].

## Results

From April 2014 to December 2017 54 patients comprising 56 bacterial meningitis episodes were included in our multicenter prospective serial sampling study (Table 1). Of these 54 patients, 35 (65%) were male and the median age was 62 years. *S. pneumoniae* was the causative pathogen in 38 of these 54 (70%) episodes. On admission, 20 of 54 (41%) presented with the classic triad of fever, neck stiffness and decreased consciousness (defined as Glasgow coma scale score of < 14) and 12 of 54 (22%) were comatose. Twelve of 54 patients (22%) died.

**Table 1:** Clinical characteristics of the 54 patients with bacterial meningitis and 38 patients with pneumococcal meningitis in the serial sampling study

Characteristics	Patients (n = 54) n/N (%)	Pneumococcal meningitis patients (n = 38) n/N (%)
Male	35/54 (65%)	22/38 (58%)
Age in years, mean (SD)	54 (21)	61 (18)
Symptoms and predisposing conditions		
Duration of symptoms < 24 h Sinusitis/otitis media Pneumonia Immunocompromised state	22/52 (42%) 16/53 (30%) 3/48 (6%) 10/53 (19%)	19/38 (50%) 13/37 (35%) 2/32 (6%) 31/38 (82%)
Clinical characteristics on admission		
Classic triad Coma Focal neurologic deficits	20/49 (41%) 12/54 (22%) 12/53 (23%)	19/34 (56%) 11/38 (29%) 10/37 (27%)
Causing pathogen		
*S. pneumoniae* *N. meningitidis* Other	38/54 (70%) 5/54 (9%) 11/54 (21%)	100% – –
Laboratory characteristics on admission**		
C-reactive protein (mg/L) Blood leukocyte count (× 10^9 cells/L) CSF leukocyte count (× 10^6 cells/L)	141 (54–267) 18.3 (11.9–23.8) 2874 (685–7877)	141 (59–293) 18.3 (11.9–26.6) 2462 (511–6170)
Standard dose of dexamethasone therapy	43/49 (88%)	32/33 (97%)
Clinical course/complications		
Circulatory shock Intensive care admission Cerebral infarction	6/47 (13%) 23/52 (44%) 6/47 (13%)	6/33 (18%) 21/35 (60%) 6/32 (19%)
Outcome at discharge		
GOS 1 GOS 3 GOS 4 GOS 5	12/54 (22%) 5/54 (9%) 7/54 (13%) 30/54 (56%)	11/38 (29%) 4/38 (11%) 4/38 (11%) 19/38 (50%)
Mortality at 3 months	14/54 (26%)	13/38 (34%)

^**^ CRP, blood leukocyte count, CSF leukocyte count were known in respectively all, 53 of 54 (pneumococcal 37 of 38) and 50 of 54 (pneumococcal 34 of 38) patients, data are median (interquartile range)

**Fig. 1:**
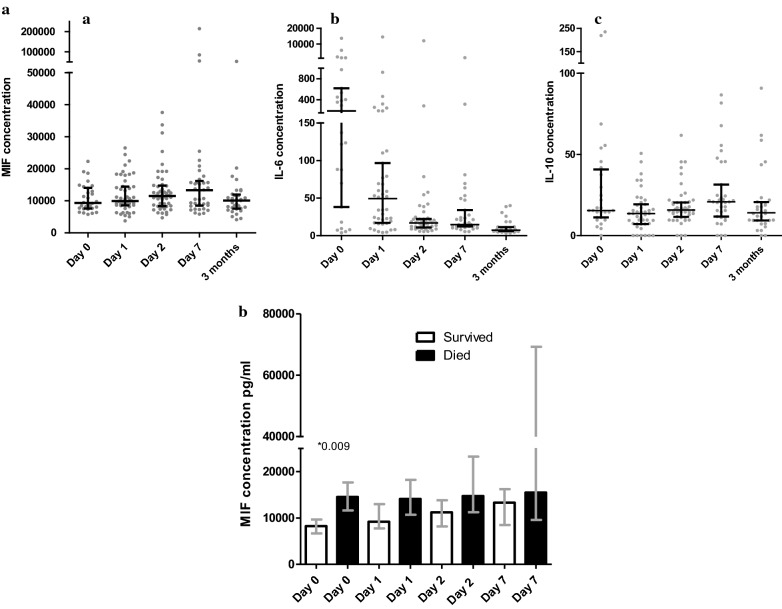
Cytokine concentrations at day 0, 1, 2 and 7 of admission and 3 months after admission. **A** Scatter plot of cytokine concentration in pg/ml of 56 bacterial meningitis episodes. Black lines are medians with interquartile ranges, **a** MIF, **b** IL-6, **c** IL-10. **B** MIF concentration in pg/ml at day 0, 1, 2 and 7 of admission of survivors versus deceased patients. White bars: survivors, black bars: patients that died during admission. Bars are presented as medians with interquartile ranges (lines in grey)

To investigate whether MIF concentrations were associated with outcome we obtained serial blood samples. Plasma samples were obtained on day 0 from 30 of 56 (54%) available episodes, on day 1 from 47 of 55 (85%) episodes, day 2 from 50 of 52 (96%) episodes, on day 7 from 37 of 56 (66%) episodes, and of patients alive after 3 months from 32 of 42 (76%) available episodes. MIF concentration during early admission (day 0) did not differ with the convalescent samples that were taken 3 months after discharge (Fig. 1a). On the contrary, for IL-6 concentrations, a clear increase was observed in the early phase of disease. In line with previous results on high-expression MIF alleles and cerebrospinal fluid concentrations [21], higher plasma concentrations of MIF on admission were associated with mortality (median MIF blood level of survivors 8,244 pg/ml [IQR 7463–11,465] vs deceased patients 14,623 pg/ml [IQR 12,949–18,020], *p* = 0.009; Fig. 1b). In the following days the same trend was visible but the larger spread of measurements led to a non-significant difference. Limiting the analysis to the 38 pneumococcal meningitis patients showed similar results, with higher plasma concentration of MIF on admission being associated with mortality (*p* = 0.039).

Subsequently, we investigated whether variability of MIF concentration after ex vivo stimulation was associated with cognitive impairment after pneumococcal meningitis in a long-term follow up study. Patient enrollment in this study have been described elsewhere [15]. For this study we included 79 patients, 1–5 years after pneumococcal meningitis, and 63 controls. Gender and age were similar between patients and controls (Table 2). As described previously, multivariate analysis of covariance showed significant differences in overall test scores of neuropsychological testing between patients and controls (*p* = 0.008) [15]. Of the cognitive domains, alertness (*p* = 0.01) and cognitive flexibility (*p* = 0.03) were most affected [15].

**Table 2:** Clinical characteristics of the 80 patients with pneumococcal meningitis and 69 controls in the follow up study

Characteristics	Patients (n = 80) n/N (%)	Controls (n = 69) n/N (%)
Male	39/80 (49%)	35/69 (51%)
Age in years*	63 (56–69)	65 (54–68)
Predisposing conditions before admission		
Duration of symptoms < 24 h Sinusitis/otitis media Pneumonia Immunocompromised state	35/79 (44%) 44/79 (55%) 10/78 (13%) 16/80 (20%)	
Clinical characteristics on admission		
Classic triad Coma Focal neurologic deficits	35/76 (44%) 4/80 (5%) 18/79 (23%)	
Laboratory characteristics on admission*		
Blood leukocyte count (× 10^9 cells/L) CSF leukocyte count (× 10^6 cells/L)	17.5 (13.3–23.6) 3492 (1298–7805)	
Standard dose of dexamethasone therapy	69/80 (86%)	
Clinical course/complications		
Seizures Circulatory shock Intensive care admission Cerebral infarction	12/76 (15%) 1/73 (1%) 26/77 (33%) 7/69 (9%)	
Outcome at discharge		
GOS 3 GOS 4 GOS 5	2/80 (3%) 17/80 (21%) 61/80 (76%)	
Years from discharge to testing*	2.5 (1.1—4.6)	

^*^Median (IQR 25–75)

Of the patients who underwent neuropsychological evaluation, whole blood or fresh isolated PBMCs were stimulated with RPMI (negative control), LTA, LPS, and two different UV-killed *S. pneumoniae* strains: D39, and ATCC 6303. As a major component of the membrane (cell wall) of all Gram positive bacteria, LTA is important for bacterial survival, growth, and pathogenicity. LPS is a major component of the outer membrane of Gram negative bacteria and plays a key role in host–pathogen interactions with the innate immune system.

**Fig. 2:**
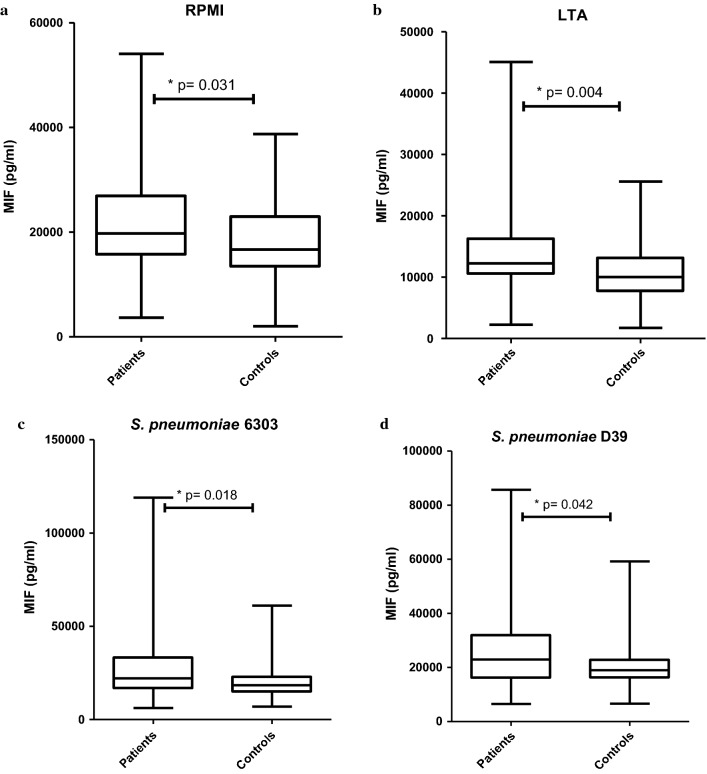
MIF concentration after 24 h whole blood stimulation of patients versus controls. Bars are boxplot with medians and interquartile ranges **a** after RPMI stimulation of WB of patients 19,459 pg/ml [IQR 15,880–26755] versus that of controls 16,182 pg/ml [IQR 13,334–23006], *p* = 0.031; **b** after LTA stimulation of WB of patients 12,461 pg/ml [IQR 10,649–16253] versus that of controls 9932 pg/ml [IQR 7718–12592], *p* = 0.009; **c** after *S. pneumoniae* 6303 stimulation of WB of patients 22,042 pg/ml [IQR 16,505–33155] versus that of controls 18,588 pg/ml [IQR 14,974–23099], *p* = 0.018; **d** after *S. pneumoniae* D39 stimulation of WB of patients 22,889 pg/ml [IQR 16,057–31820] versus that of controls 18,805 pg/ml [IQR 16,063–22865], *p* = 0.042. See Additional file 1: Figs. 2.1 and 2.2 for IL-6 and IL-10 concentrations after 24 h WB stimulation of patients versus controls (no significant differences)

**Fig. 3:**
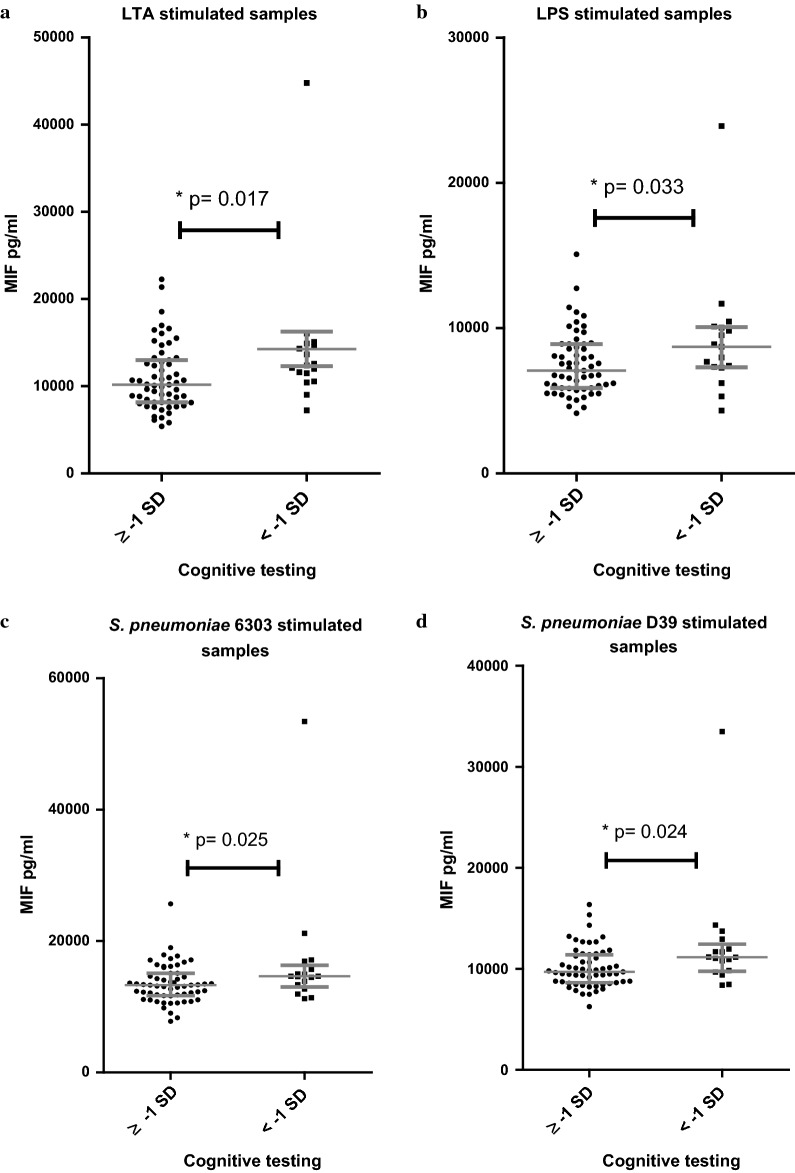
MIF concentration after 24 h PBMC stimulation versus performance on cognitive testing. MIF concentration in pg/ml after 24 h PBMC stimulation in groups with good performance on cognitive testing (test score ≥ − 1SD) and worse performance on cognitive testing (test score < − 1 SD). **a** After stimulation with LTA. **b** After stimulation with LPS. **c** After stimulation with *S. pneumoniae* 6306. **d** After stimulation with *S. pneumoniae* D39. Grey lines are medians and interquartile ranges. See Additional file 1: Figs. 3.1 and 3.2 for IL-6 and IL-10 concentrations after 24 h PBMC stimulation in groups divided on performance on cognitive testing (no significant differences)

IL-6 and IL-10 concentration were increased 24 h after ex vivo stimulation with LTA, LPS and both pneumococcal strains, as compared to the negative control samples with RPMI (Additional file 1: Fig. 1b and c). MIF concentration was not increased after whole blood and PBMC stimulation with LTA, LPS or pneumococci, compared to negative control samples with RPMI (Additional file 1: Fig. 1a). However, in whole blood stimulation experiments patients showed higher MIF responses than control subjects for all stimuli (respectively *p* = 0.031, *p* = 0.004, *p* = 0.018, *p* = 0.042, Fig. 2). Whole blood stimulation showed no differences in MIF response between patients and control subjects, but MIF concentrations after PBMC stimulation were higher in patients with a worse performance on the most affected cognitive domain alertness (Fig. 3).

## Discussion

Our study confirms the role of MIF in poor disease outcome in the acute phase of pneumococcal meningitis. Our findings are in line with a previous study that described high-expression MIF alleles to be associated with disease severity and death in patients with pneumococcal meningitis. We found that higher plasma MIF concentrations during the early phase of disease were predictive for mortality in bacterial meningitis. Our findings are consistent with the harmful consequences of robust pro-inflammatory cytokine responses on brain edema and neuronal damage in the course of bacterial meningitis [1]. MIF has also shown to be markedly and persistently upregulated and to be associated with increased disease severity and early death in patients with sepsis [24, 25]. Administration of recombinant MIF protein in a murine sepsis model increased mortality following LPS administration [26]. Several experimental sepsis studies in mice showed that the neutralization of MIF reduced pro-inflammatory cytokine production and organ injury, and thereby increased the survival rate [21, 27, 28]. Therefore, MIF modulation is an interesting adjunctive therapy to improve outcome of pneumococcal meningitis.

Our study shows that individuals with an increased pro-inflammatory response consisting of a higher MIF expression after PBMC stimulation, are at risk for worse cognitive functioning. Previous studies showed that pneumococcal meningitis patients were at risk to develop cognitive impairment [13–16, 29]. The pathophysiology of cognitive impairment after pneumococcal meningitis is currently unknown. In a prospective cohort study, baseline data, including clinical characteristics, and cytobiochemical parameters of blood and cerebrospinal fluid between patients with or without cognitive impairment after meningitis were similar [14]. In patients with clinical pre-dementia disease stage, MIF has been associated with biomarkers of Alzheimer’s disease pathology and predicted cognitive impairment [30]. MIF cerebrospinal fluid (CSF) levels in even moderately cognitively impaired subjects were higher compared to participants with normal cognition [30]. Experimental studies showed that MIF deficient mice had reduced astrocyte activation and tau hyperphosphorylation in Alzheimer’s disease models [31].

High MIF responsiveness may persist for a long time after the acute disease. Blood MIF levels on admission were not different than those among survivors 3 months after infection, although we did not test MIF levels in control subjects. However, even years after the disease, patients had higher MIF responsiveness on infectious stimuli compared to controls. A study with patients suffering from sepsis showed consistent results with higher ex vivo MIF release by PBMCs in patients versus healthy control subjects [32]. Furthermore, murine models of sepsis have shown low-grade brain inflammation persists after recovering from sepsis, suggesting a severe infection is able to induce in microglia a primed-like state [33, 34]. The persistent brain inflammation was associated with increased levels of amyloid-beta peptide and long-term cognitive deficits in sepsis survivors [33]. Likewise in patient studies it is known that severe sepsis in the older population is independently associated with substantial and persistent new cognitive impairments [35]. Although with this study we cannot prove a causal relationship, we hypothesize that prolonged MIF upregulation contributes to the cognitive impairments of survivors of pneumococcal meningitis. This hypothesis is strengthened by studies showing increased MIF production is associated with Alzheimer disease and mild cognitive impairment suggesting that MIF is involved in the neuro-inflammatory process occurring in cognitive decline [36, 37]. One study has shown MIF can bind to the amyloid protein, possibly leading to accumulation of amyloid-beta in Alzheimer disease [38].

Our study has several limitations. The most important limitation is selection bias. First, we did not sample all patients in the acute phase of disease. Samples from early time points were missed because of informed consent procedures. At later time points some patients had died. These two factors led to selection bias leading to underrepresentation of the most severely ill patients. This might have caused an underestimation of the predictive effect of MIF concentrations. Second, all patients in our prospective study underwent lumbar puncture. Since lumbar puncture in some cases cannot be done in the most severe patients, this may also have led to an underestimation of the rate of unfavorable outcome and death in our cohort. Third, the patients in the follow up study were a selected group of patients with relatively good condition after disease, which could have underestimated the rate of cognitive impairment, decreasing the study power to detect meaningful associations between individual MIF responsiveness and cognitive impairment. Another limitation is that we did not use specific cell subpopulations for our ex vivo stimulation experiments. Stimulation of specific cell population would likely have increased our study power, providing more specific information [39]. Interestingly, stimulation experiments showed consistent results for all stimuli which may suggest that our results are robust—at least between different stimuli.

## Conclusions

Our study shows that high MIF concentrations in the early phase of acute bacterial meningitis predict poor outcome of disease. Furthermore, we found associations between high MIF levels and occurrence of cognitive impairment, suggesting MIF contributes to cognitive impairments in pneumococcal meningitis. Both results suggest MIF modulating therapy could be an interesting new target to influence outcome of pneumococcal meningitis.

## Supplementary Information

**Additional file 1** Suplementary material.

## Abbreviations

COGBAT: Cognitive basic assessment test set
CSF: Cerebrospinal fluid
IQR: Interquartile range
LPS: Lipopolysaccharide
LTA: Lipoteichoic acid
MIF: Macrophage migration inhibitory factor
MOI: Multiplicity of infection
PBMC: Peripheral blood mononuclear cell
RPMI: Roswell Park Memorial Institute
UV: Ultraviolet
VTS: Vienna test system
WB: Whole blood
WMO: Dutch medical research involving human subjects
