# Supplementary material for: Individual responsiveness of macrophage migration inhibitory factor predicts long-term cognitive impairment after bacterial meningitis
Source: Acta Neuropathol Commun. 2021 Jan 6;9:4. doi: 10.1186/s40478-020-01100-7 (PMC7789269; doi:10.1186/s40478-020-01100-7)
Supplement: Supplementary file 1 — Additional file 1 Suplementary material. [file 40478_2020_1100_MOESM1_ESM.docx]

**SUPPLEMENTARY FIGURES**

**Supplementary figure 1 - Cytokine levels of patients and controls after 24 hour WB and PBMC stimulation**

1a

1b

1c

Supplementary figure 1 – Scatter plots of cytokine concentration of patients and controls after 24 hour WB (n=141) and PBMC (n=140) stimulation with RPMI, LTA, LPS*, S. pneumoniae* 6303 and *S. pneumoniae* D39. Bars are medians with interquartile ranges. 1a) MIF concentration in pg/ml. 1b) IL-6 concentration in pg/ml. 1c. IL-10 concentration in pg/ml.

**Supplementary figure 2.1 – IL-6 concentration after 24 hour whole blood stimulation of patients versus controls**

Supplementary figure 2.1 – IL-6 concentration in pg/ml. Bars are boxplot with medians and interquartile ranges. **2a)** after stimulation with RPMI no significant differences. **2b)** after stimulation with LTA no significant differences. **2c)** after stimulation with *S. pneumoniae* 6303 no significant differences. **2d)** after stimulation with *S. pneumoniae* D39 no significant differences.

**Supplementary figure 2.2 – IL-10 concentration after 24 hour whole blood stimulation of patients versus controls**

Supplementary figure 2.2 – IL-10 concentration in pg/ml. Bars are boxplot with medians and interquartile ranges. **2a)** after stimulation with RPMI no significant differences. **2b)** after stimulation with LTA no significant differences. **2c)** after stimulation with *S. pneumoniae* 6303 no significant differences. **2d)** after stimulation with *S. pneumoniae* D39 no significant differences.

**Supplementary figure 3.1** **– IL-6 and 3.2 – IL-10 concentration after 24 hour PBMC stimulation versus performance on cognitive testing**

3.1 a 3.1 b

3.1 c3.1 d

3.2 a 3.2 b

3.2 c 3.2 d

Supplementary figure 3.1 – IL-6 concentration in pg/ml after 24 hour PBMC stimulation. 3.2 IL-10 concentration in pg/ml after 24 hour PBMC stimulation. Groups are divided between good performance on cognitive testing (test score ≥ -1SD) and worse performance on cognitive testing (test score < -1 SD). a) After stimulation with LTA. b) After stimulation with LPS. c) After stimulation with *S. pneumoniae* 6306. d) After stimulation with *S. pneumoniae* D39. Grey lines are medians and interquartile ranges.
